# Crystal structure of *S*-*n*-octyl 3-(1-phenyl­ethyl­idene)di­thio­carbazate and of its bis-chelated nickel(II) complex

**DOI:** 10.1107/S2056989023009726

**Published:** 2023-11-14

**Authors:** Sultana Shakila Khan, Md. Belayet Hossain Howlader, Md. Chanmiya Sheikh, Ryuta Miyatake, Ennio Zangrando, Md. Rezaul Haque Ansary

**Affiliations:** a Department of Chemistry, Rajshahi University, Rajshahi-6205, Bangladesh; b Department of Applied Science, Faculty of Science, Okayama University of Science, Japan; c Center for Environmental Conservation and Research Safety, University of Toyama, 3190 Gofuku, Toyama, 930-8555, Japan; d Department of Chemical and Pharmaceutical Sciences, University of Trieste, Italy; University of Durham, United Kingdom

**Keywords:** crystal structure, di­thio­carbazate ligand, Ni^II^ complex, *cis* configuration complex, octyl alkyl chain

## Abstract

The nitro­gen–sulfur Schiff base proligand *S*-*n*-octyl 3-(1-phenyl­ethyl­idene)di­thio­carbazate, C_17_H_26_N_2_S_2_ (H*L*), was prepared by reaction of *S*-octyl di­thio­carbamate with aceto­phenone. Treatment of H*L* with nickel acetate yielded the complex bis­[*S*-*n*-octyl 3-(1-phenyl­ethyl­idene)di­thio­carbazato]nickel(II), [Ni(C_17_H_25_N_2_S_2_)_2_] (Ni*L*_2_), which was shown to adopt a tetra­hedrally distorted *cis*-square-planar coordination geometry, with the NiSN planes of the two ligands forming a dihedral angle of 21.66 (6)°. Changes in the geometry of the *L* ligand upon chelation of Ni^2+^ are described, involving a *ca* 180° rotation around the N(azomethine)—C(thiol­ate) bond.

## Abstract 2

A bis-chelated mononuclear nickel(II) complex with a di­thio­carbazate ligand bearing a long saturated alkyl chain exhibits a distorted *cis* square planar coordination of the metal with two ligands conformationally different from the proligand.

## 1. Chemical context

Bidentate Schiff bases of *S*-methyl di­thio­carbazate (SMDTC) or *S*-benzyl di­thio­carbaza­tes (SBDTC) and their bivalent metal complexes have received considerable attention in the field of medical science for their biological activities (Cavalcante *et al.*, 2019 ▸; Chan *et al.*, 2008 ▸; Chew *et al.*, 2004 ▸; Crouse *et al.*, 2004 ▸; How *et al.*, 2008 ▸; Yang *et al.*, 2020 ▸). As part of our ongoing inter­est in S-containing Schiff bases and the corres­ponding metal complexes, we report herein on the structure of a ligand mol­ecule having an octyl alkyl chain and of its bis-chelated nickel complex. 
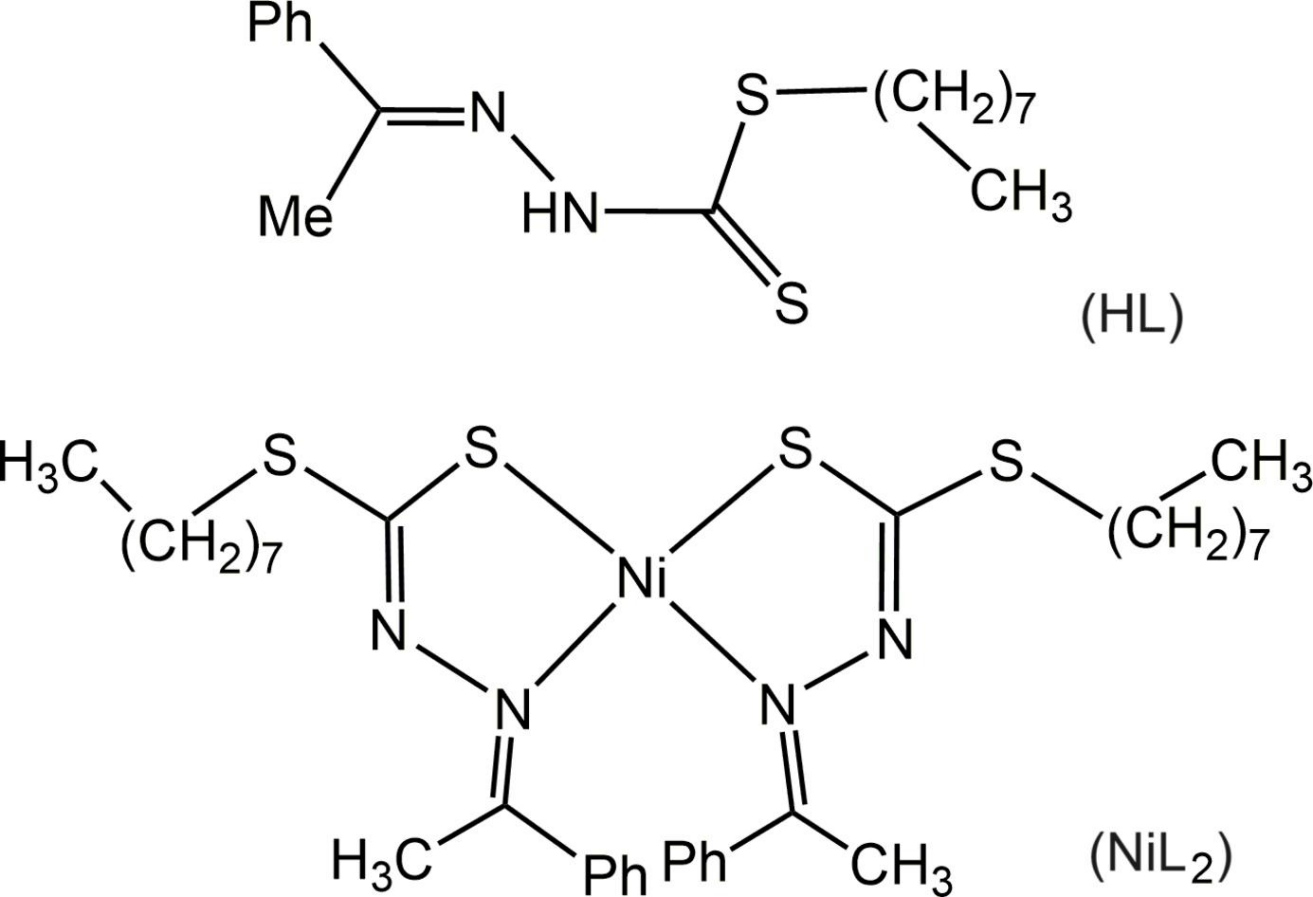


## 2. Structural commentary

**Figure 1:**
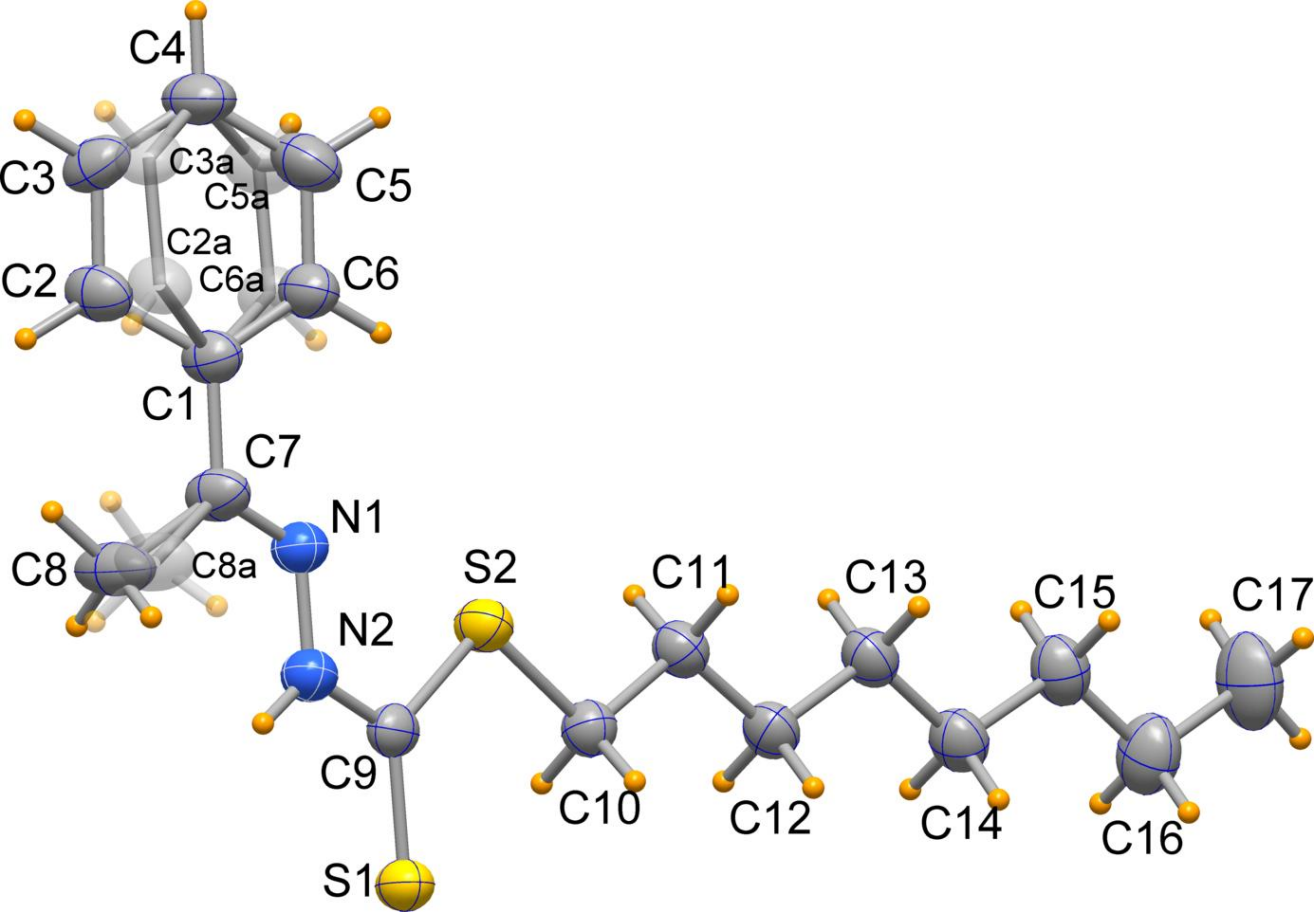
Mol­ecular structure of H*L*. Atomic displacement ellipsoids are drawn at the 50% probability level.

The H*L* proligand crystallizes in its thione tautomeric form (Fig. 1 ▸). The β-N atom (N1) and the thio­keto atom S1 are located in *trans* positions with respect to the C9—N2 bond, as has been observed in other similar di­thio­carbazate species (Begum *et al.*, 2015 ▸). The phenyl ring is disordered with equal probability between two orientations, differing by a dihedral angle of 42.2 (3)°. The adjacent methyl group C8H_3_ is likewise disordered, the directions of the C7—C8 bond differing by 23.1 (1)°. While there can be some ambiguity on how the disorder of the phenyl and methyl groups is correlated *intra*mol­ecularly, we suggest that the near-eclipsed conformation about the C1—C7 bond (as shown here) is more likely than the alternative one (twisted by 31 or 38°), because the former conformation was typically observed in previously studied compounds of ArC(Me)=NNHC(=S)S*R* type, where Ar is a phenyl group or a phenyl substituted in a *meta* or *para* (but not *ortho*) position (see Section 4).

**Figure 2:**
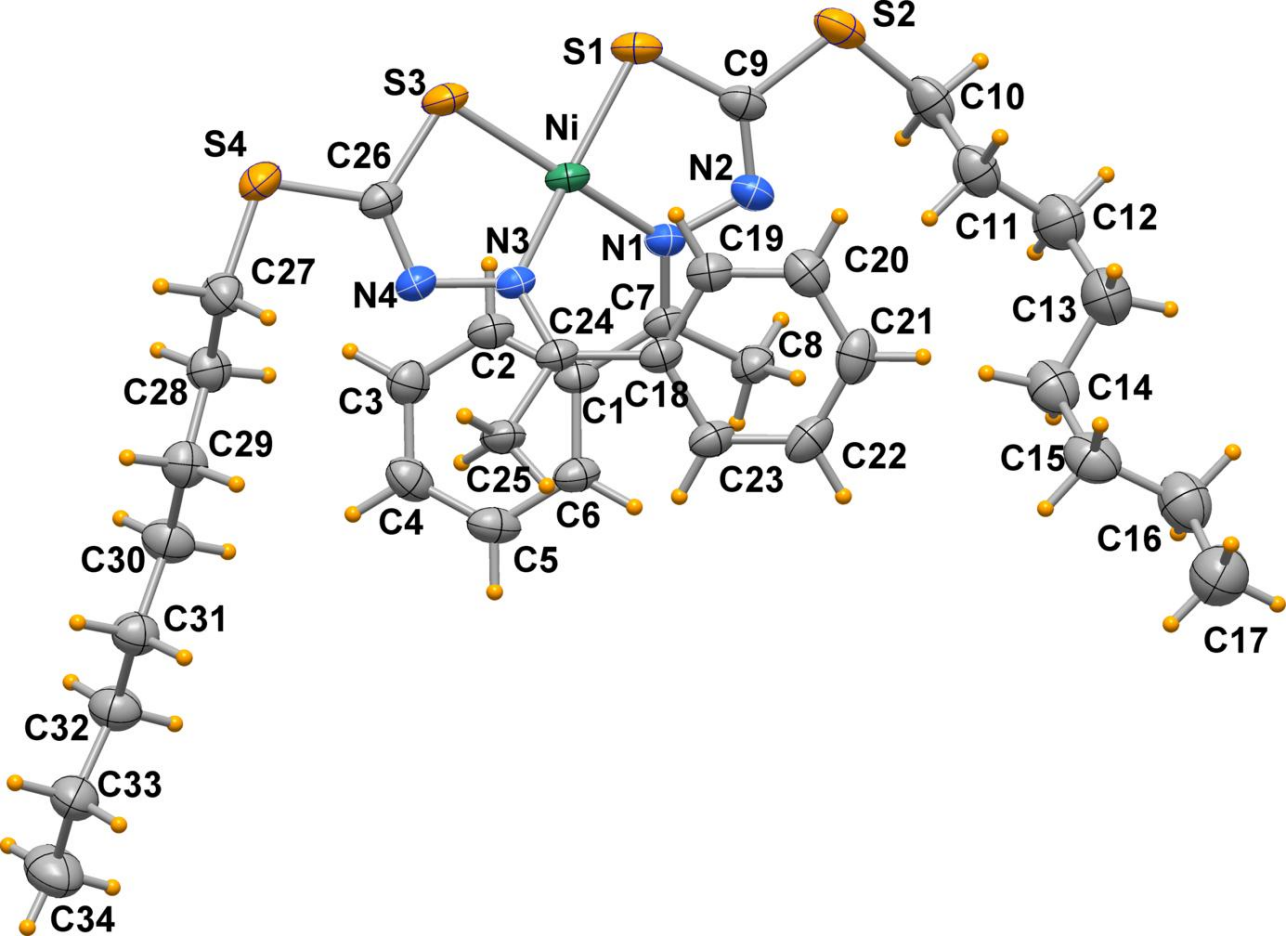
Mol­ecular structure of the Ni*L*_2_ complex. Atomic displacement ellipsoids are drawn at the 50% probability level.

In the Ni*L*_2_ complex (Fig. 2 ▸) the two Schiff bases *L* in their deprotonated imino thiol­ate form, coordinate the metal through the β-nitro­gen atoms, N1 or N3, and the thiol­ate sulfur, S1 or S3, respectively, in a *cis*-square-planar configuration which is tetra­hedrally distorted in order to avoid steric clashes between the phenyl rings. The dihedral angle formed by the NiNS planes of the two five-membered chelate rings is thus 21.66 (6)°. The Ni—S bond distances of 2.1506 (6) and 2.1573 (6) Å are similar, as are the Ni—N ones of 1.9392 (16) and 1.9318 (15) Å. The orientation of the phenyl groups is such that their *ortho* hydrogen atoms are located in apical positions above and below the metal centre, with the Ni⋯H separations of *ca* 2.6 Å indicating possible non-covalent inter­actions.

Some important geometrical changes are observed in the ligand upon coordination, the most significant being the elongation of the S1=C9 bond of 1.669 (3) Å in H*L* to the essentially single bonds of 1.738 (2) Å in the complex, thus validating the coordination with deprotonated thiol­ate sulfur atom. Correspondingly the N2—C9 bond of 1.340 (4) Å in H*L* shortens to essentially double bonds of 1.293 (3) and 1.290 (2) Å in the complex, while the N1—N2 bond length of 1.377 (4) Å is slightly elongated in the complex [to 1.414 (2) and 1.417 (2) Å, see the supporting information]. These parameters agree with those in previously reported Ni^II^ complexes with similar ligands (Begum *et al.*, 2016 ▸, 2017 ▸, 2020 ▸, 2023 ▸; Howlader *et al.*, 2015 ▸; Islam *et al.*, 2014 ▸; Khan *et al.*, 2023 ▸; Zangrando *et al.*, 2015 ▸). Upon coordination the ligand *L* undergoes a rotation of *ca*. 180° about the N2—C9 bond to chelate the metal through the N and S donors.

The *n*-octyl chain in H*L* has an extended all-*trans* conformation and is practically coplanar with the di­thio­carbazate moiety. In the complex, one *n*-octyl chain (C27 to C34) also adopts an all-*trans* conformation (although tilted out of the coordination plane), while the other one is ‘kinked’ due to the *gauche* conformation about the C13—C14 bond.

An analysis of di­thio­carbazate ligands in bis-chelated Ni and Cu complexes of *cis* and *trans* arrangement was reported by us earlier (Begum *et al.*, 2020 ▸). Among the Ni^II^ complexes with di­thio­carbazate Schiff base *N*,*S*-ligands having long alkyl chains, the *cis* configuration was observed in derivatives with a phenyl­ethyl­idene fragment bound at N1 (Zangrando *et al.*, 2015 ▸; Begum *et al.*, 2020 ▸), as in the present complex.

## 3. Supra­molecular features

**Figure 3:**
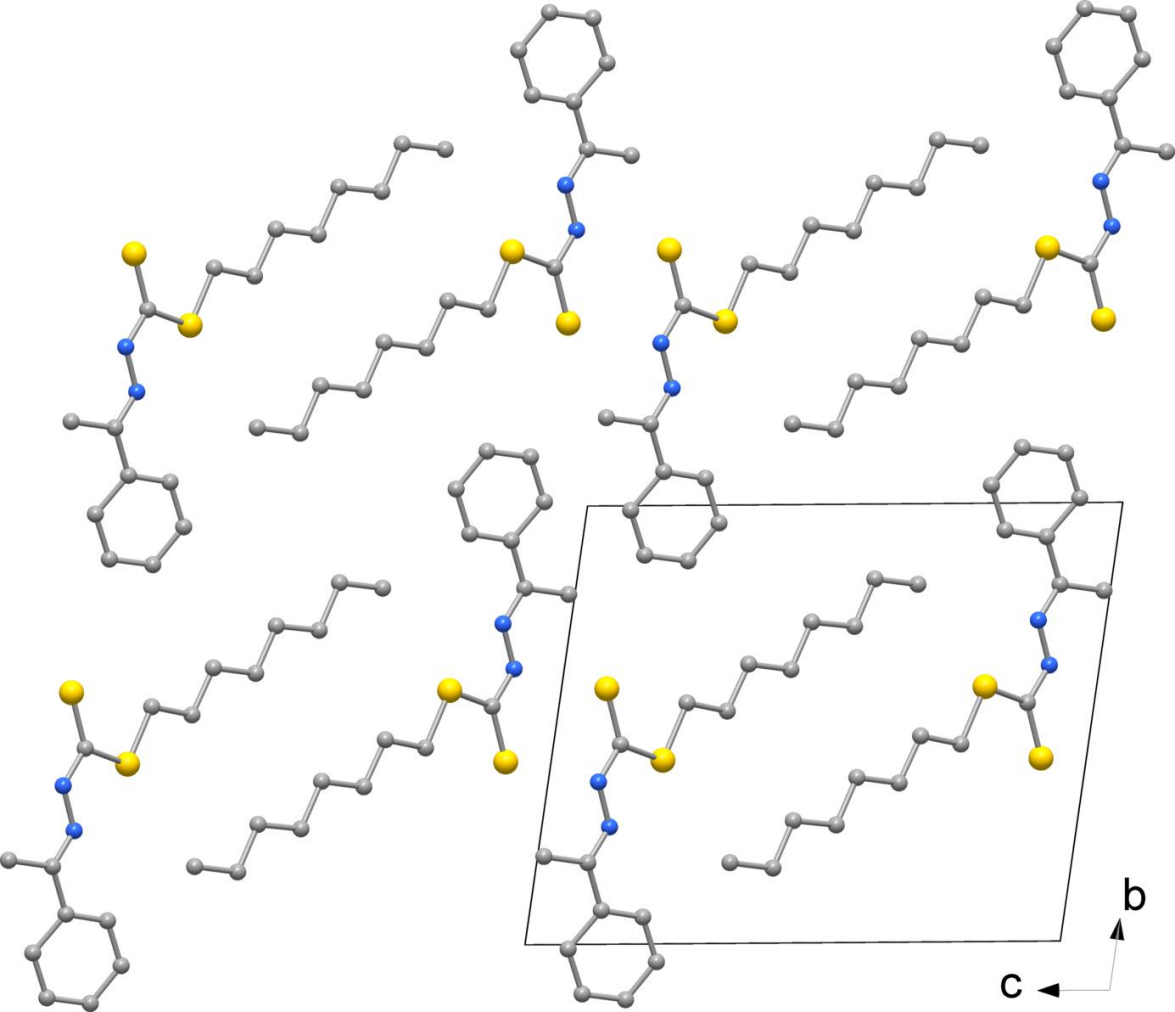
Crystal packing of H*L* viewed down the *a* axis (H atoms omitted and only one orientation of the disordered phenyl rings is shown for clarity).

The crystal pacing of H*L* is shown in Fig. 3 ▸. The crystal structure contains segregated regions of polar di­thio­carbazate moieties, hydro­phobic alkyl chains and aromatic phenyl groups.

It is noteworthy that there are some sterically impossible short distances between symmetry-related positions of the disordered phenyl rings, *e.g*. C2⋯C2 of 2.72 Å between mol­ecules related by an inversion centre, and C2*A*⋯C5*A* of 2.82 Å between mol­ecules related by the translation **a**. Obviously, these orientations cannot be adopted by adjacent mol­ecules simultaneously and the respective symmetry operations are locally spurious.

**Figure 4:**
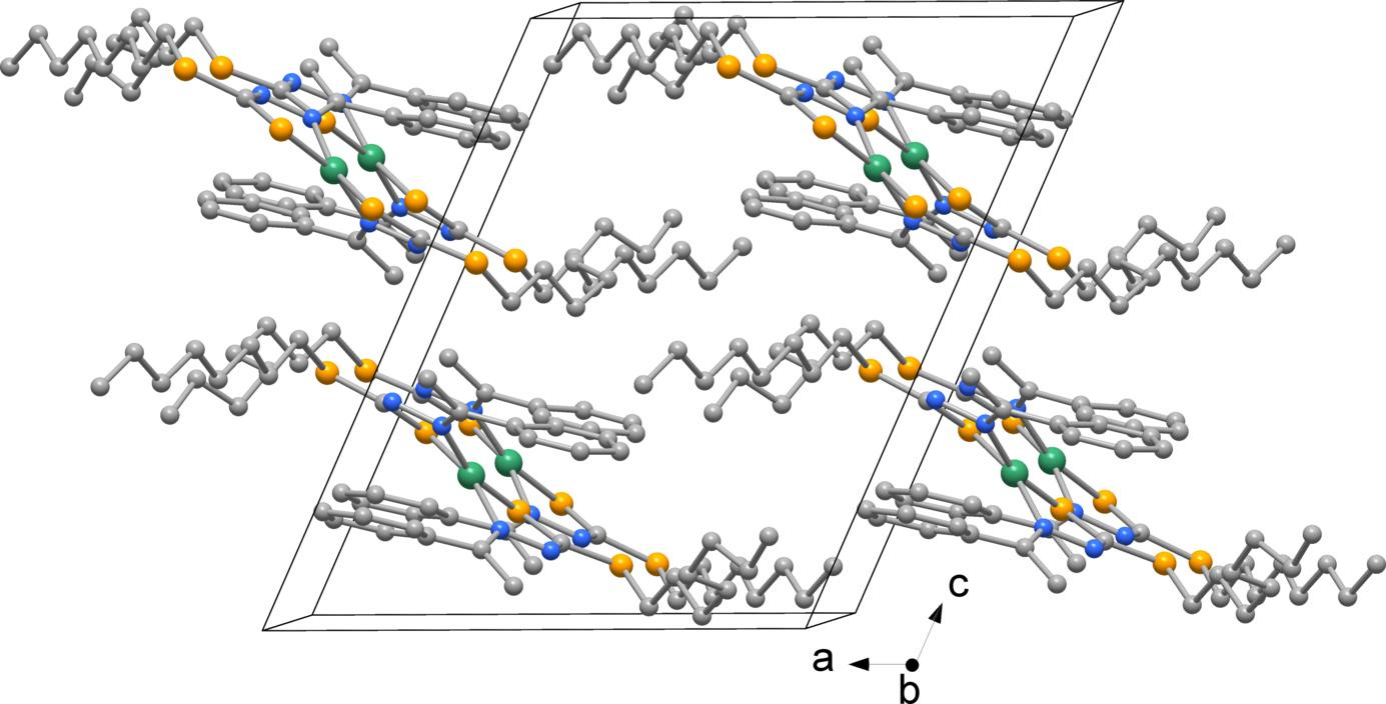
View of the crystal packing of the Ni*L*_2_ complex down the *b* axis (H atoms not shown for clarity).

The packing of Ni*L*_2_ is shown in Fig. 4 ▸; the *cis* coordination does not allow the mol­ecules to stack at short distances as observed for *trans* square-planar species with analogous ligands (Howlader *et al.*, 2015 ▸; Begum *et al.*, 2016 ▸).

## 4. Database survey

Numerous Ni^II^ complexes with di­thio­carbazate ligands have been reported from these laboratories (Begum *et al.*, 2016 ▸, 2017 ▸, 2020 ▸, 2023 ▸; Howlader *et al.*, 2015 ▸; Islam *et al.*, 2014 ▸; Khan *et al.*, 2023 ▸; Zangrando *et al.*, 2015 ▸; CSD refcodes = JUYCAJ, WEGKEB, TILVUJ, PICMOH, LUBYAK, MIXRAO, MIMKIG and LUBNON, respectively).

Reported structures of the ArC(Me)=NNHC(=S)S*R*-type compounds include GUMJUV (Bin Break *et al.*, 2013 ▸), HUXNAS (Boshaala, Flörke *et al.*, 2021 ▸), LOBZUY (Shan *et al.*, 2008 ▸), LUBNIH (Zagrando *et al.*, 2015), OKIVUB (Nanjundan *et al.*, 2016 ▸), PIFMAT (How *et al.*, 2007 ▸), UWATOD (Flörke & Boshaala, 2016 ▸) and UWAVEV (Boshaala, Said *et al.*, 2021 ▸). All these mol­ecules have broadly the same configuration as H*L*. The ArCMe skeleton is usually practically planar, the Ar and adjacent Me groups deviating from the eclipsed orientation by less than 5°, except in GUMJUV (14.5°), PIFMAT (20.4°) and one of the three independent mol­ecules in the structure of OKIVUB (10.8°).

## 5. Synthesis and crystallization

**Proligand H** ***L***: 30 mL of an ethano­lic solution of KOH (2.81 g, 0.05 mol) was mixed with hydrazine hydrate (2.50 g, 0.05 mol, 99%) and stirred at 273 K. To this solution carbon di­sulfide (3.81 g, 0.05 mol) was added dropwise with constant stirring for 1 h. Then 1-bromo­octane (9.65 g, 0.05 mol) was added dropwise with vigorous stirring at 273 K for 1 h. Finally, 2 mL of an ethano­lic solution of aceto­phenone (6.00 g, 0.05 mol) were added and the resulting mixture was refluxed for 30 min. The hot mixture was filtered and the filtrate was cooled to 273 K giving a precipitate of Ni*L*_2_, which was recrystallized from ethanol at room temperature, filtered off and dried in a vacuum desiccator over anhydrous CaCl_2_. Colourless plate-shaped crystals suitable for X-ray diffraction were obtained by slow evaporation from a mixture of ethanol and methanol (2:1, *v*/*v*) after 15 days. The physical and spectroscopic data are as follows:

Colourless crystalline, yield 78%, m.p. 335–336 K. FT–IR data (KBr disc, cm^−1^): ν(N—H) 3232, ν(C—H, alk­yl) 2958, 2922, ν(C=N) 1639, ν(C=C) 1607, ν(C=S) 1060. ^1^H NMR (400 MHz, CDCl_3_, ppm) δ: 9.91 (*s*, 1H, NH), 7.85 (*d*, 2H, C-2, 6), 7.41 (*t*, 3H, C-3, 4, 5), 3.31 (*t*, 2H, C-10, –SCH_2_,), 2.33 [*s*, 3H, C-8, CH_3_—C(C)=N], 1.75 (*p*, 2H, C-11), 1.45 (*p*, 2H, C-12), 1.34–1.26 (*m*, 8H, C-13, 14, 15, 16), 0.90 (*t*, 3H, C-17, CH_3_). HRMS (FAB) Calculated for C_17_H_26_N_2_S_2_ [*M*+H]^+^: 323.16102, found [*M*+H]^+^: 323.16128.

**Ni complex**: Ni(CH_3_COO)_2_·4H_2_O (0.12 g, 0.5 mmol) in 10 mL of methanol was added to a solution of (0.322 g, 1.0 mmol) in 30 mL of methanol. The resulting mixture was stirred at room temperature for 4 h. A shiny green precipitate formed, was filtered off, washed with methanol and dried *in vacuo* over anhydrous CaCl_2_. Green needle-shaped crystals suitable for X-ray diffraction were obtained by slow evaporation of the compound from a mixture of chloro­form and aceto­nitrile (5:1, *v*/*v*) after 19 days. The physical and spectroscopic data of the compound are as follows:

Green crystalline, Yield: 74%; m. p. 408–409 K. FT–IR data (KBr disc, cm^−1^): ν(C—H, alk­yl) 2949, 2924, ν(C=N—N=C) 1599, ν(C=C) 1562. ^1^H NMR (400 MHz, CDCl_3_, ppm) δ: 7.56 (*t*, 2×3H, C-3, 4, 5), 7.47 (*d*, 2×2H, C-2, 6), 2.91 (*t*, 2×2H, C-10, –SCH_2_,), 1.87 [*s*, 2×3H, C-8, CH_3_—C(C)=N], 1.67 (*p*, 2×2H, C-11), 1.38 (*p*, 2×2H, C-12), 1.33–1.27 (*m*, 2×8H, C-13, 14, 15, 16), 0.89 (*t*, 2×3H, C-17, CH_3_). UV–Vis spectrum in CHCl_3_ [λ_max_ nm, ɛ_max_ *M* ^−1^ cm^−1^]: 222, 35240; 280, 57000; and 384, 12420. HRMS (FAB) Calculated for C_34_H_50_N_4_NiS_4_ [*M*+H]^+^: 701.23445, found [*M*+H]^+^: 701.23420.

## 6. Refinement details

Crystal data, data collection and structure refinement details are summarized in Table 1 ▸. The phenyl ring of the uncoordinated ligand was found disordered over two positions with equal (0.5) occupancies. All H atoms were geometrically located with exception of that at N2 in the free ligand which was freely refined.

## Supplementary Material

Crystal structure: contains datablock(s) HL, NiL2, global. DOI: 10.1107/S2056989023009726/zv2030sup1.cif

Structure factors: contains datablock(s) NiL2. DOI: 10.1107/S2056989023009726/zv2030NiL2sup3.hkl

CCDC references: 2254903, 2254853

Additional supporting information:  crystallographic information; 3D view; checkCIF report

## Figures and Tables

**Table 1:** Experimental details

	H*L*	Ni*L*_2_
Crystal data		
Chemical formula	C_17_H_26_N_2_S_2_	[Ni(C_17_H_25_N_2_S_2_)_2_]
*M*_r_	322.52	701.73
Crystal system, space group	Triclinic, *P* 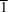	Monoclinic, *P*2_1_/*n*
Temperature (K)	173	173
*a*, *b*, *c* (Å)	4.9925 (6), 12.4283 (16), 15.0643 (19)	13.6399 (3), 17.6532 (5), 16.7596 (3)
α, β, γ (°)	98.420 (7), 94.302 (7), 91.150 (6)	90, 114.000 (8), 90
*V* (Å^3^)	921.6 (2)	3686.6 (3)
*Z*	2	4
Radiation type	Mo *K*α	Mo *K*α
μ (mm^−1^)	0.29	0.78
Crystal size (mm)	0.19 × 0.10 × 0.07	0.27 × 0.09 × 0.03

Data collection		
Diffractometer	Rigaku R-AXIS RAPID	Rigaku R-AXIS RAPID
Absorption correction	Multi-scan (*ABSCOR*; Higashi, 1995 ▸)	Multi-scan (*ABSCOR*; Higashi, 1995 ▸)
*T*_min_, *T*_max_	0.410, 0.980	0.737, 0.977
No. of measured, independent and observed [*I* > 2σ(*I*)] reflections	8449, 4181, 3093	35807, 8419, 6674
*R*_int_	0.037	0.038
(sin θ/λ)_max_ (Å^−1^)	0.649	0.649

Refinement		
*R*[*F* ^2^ > 2σ(*F* ^2^)], *wR*(*F* ^2^), *S*	0.071, 0.196, 1.08	0.042, 0.096, 1.04
No. of reflections	4181	8419
No. of parameters	242	392
H-atom treatment	H atoms treated by a mixture of independent and constrained refinement	H-atom parameters constrained
Δρ_max_, Δρ_min_ (e Å^−3^)	0.55, −0.32	0.60, −0.25

Computer programs: *RAPID-AUTO* (Rigaku, 2018 ▸), *SHELXT2018/2* (Sheldrick, 2015*a* ▸), *SHELXL2019/2* (Sheldrick, 2015*b* ▸), *DIAMOND* (Brandenburg, 1999 ▸) and *WinGX* (Farrugia, 2012 ▸).

## supplementary crystallographic information

### S-n-Octyl 3-(1-phenylethylidene)dithiocarbazate (HL) Crystal data

**Table:**

C_17_H_26_N_2_S_2_	*Z* = 2
*M**_r_* = 322.52	*F*(000) = 348
Triclinic, *P*1	*D*_x_ = 1.162 Mg m^−^^3^
*a* = 4.9925 (6) Å	Mo *K*α radiation, λ = 0.71075 Å
*b* = 12.4283 (16) Å	Cell parameters from 6282 reflections
*c* = 15.0643 (19) Å	θ = 2.0–27.5°
α = 98.420 (7)°	µ = 0.28 mm^−^^1^
β = 94.302 (7)°	*T* = 173 K
γ = 91.150 (6)°	Plate, colorless
*V* = 921.6 (2) Å^3^	0.19 × 0.10 × 0.07 mm

### S-n-Octyl 3-(1-phenylethylidene)dithiocarbazate (HL) Data collection

**Table:**

Rigaku R-AXIS RAPID diffractometer	3093 reflections with *I* > 2σ(*I*)
ω scans	*R*_int_ = 0.037
Absorption correction: multi-scan (ABSCOR; Higashi, 1995)	θ_max_ = 27.5°, θ_min_ = 2.3°
*T*_min_ = 0.410, *T*_max_ = 0.980	*h* = −6→5
8449 measured reflections	*k* = −16→16
4181 independent reflections	*l* = −19→19

### S-n-Octyl 3-(1-phenylethylidene)dithiocarbazate (HL) Refinement

**Table:**

Refinement on *F*^2^	0 restraints
Least-squares matrix: full	Hydrogen site location: mixed
*R*[*F*^2^ > 2σ(*F*^2^)] = 0.071	H atoms treated by a mixture of independent and constrained refinement
*wR*(*F*^2^) = 0.196	*w* = 1/[σ^2^(*F*_o_^2^) + (0.0649*P*)^2^ + 1.6447*P*] where *P* = (*F*_o_^2^ + 2*F*_c_^2^)/3
*S* = 1.08	(Δ/σ)_max_ < 0.001
4181 reflections	Δρ_max_ = 0.55 e Å^−^^3^
242 parameters	Δρ_min_ = −0.32 e Å^−^^3^

### S-n-Octyl 3-(1-phenylethylidene)dithiocarbazate (HL) Special details

**Table:**

Geometry. All esds (except the esd in the dihedral angle between two l.s. planes) are estimated using the full covariance matrix. The cell esds are taken into account individually in the estimation of esds in distances, angles and torsion angles; correlations between esds in cell parameters are only used when they are defined by crystal symmetry. An approximate (isotropic) treatment of cell esds is used for estimating esds involving l.s. planes.

### S-n-Octyl 3-(1-phenylethylidene)dithiocarbazate (HL) Fractional atomic coordinates and isotropic or equivalent isotropic displacement parameters (Å^2^)

**Table:**

	*x*	*y*	*z*	*U*_iso_*/*U*_eq_	Occ. (<1)
N1	−0.1043 (6)	0.7323 (2)	0.1266 (2)	0.0401 (7)	
N2	−0.2090 (6)	0.6296 (2)	0.0940 (2)	0.0409 (7)	
H2N	−0.344 (7)	0.616 (3)	0.053 (2)	0.031 (9)*	
S1	−0.20157 (19)	0.41740 (7)	0.08485 (6)	0.0432 (3)	
S2	0.16745 (18)	0.58140 (7)	0.20863 (6)	0.0431 (3)	
C1	−0.0902 (7)	0.9225 (3)	0.1334 (3)	0.0423 (8)	
C2A	−0.2308 (19)	1.0181 (6)	0.1290 (6)	0.0504 (19)	0.5
H2A	−0.412706	1.013940	0.104862	0.060*	0.5
C3A	−0.107 (2)	1.1189 (7)	0.1597 (7)	0.056 (2)	0.5
H3A	−0.196708	1.183166	0.149810	0.067*	0.5
C2	−0.1086 (16)	1.0104 (6)	0.0810 (6)	0.0461 (17)	0.5
H2	−0.190437	0.998522	0.021250	0.055*	0.5
C3	−0.0052 (18)	1.1128 (7)	0.1190 (7)	0.049 (2)	0.5
H3	−0.031247	1.173157	0.087547	0.058*	0.5
C4	0.1357 (10)	1.1268 (3)	0.2027 (3)	0.0619 (12)	
H4	0.221191	1.194944	0.226559	0.074*	
C5A	0.283 (2)	1.0330 (8)	0.2134 (7)	0.060 (2)	0.5
H5A	0.460126	1.039291	0.241511	0.072*	0.5
C6A	0.1629 (19)	0.9312 (8)	0.1820 (7)	0.049 (2)	0.5
H6A	0.252208	0.867290	0.193560	0.059*	0.5
C5	0.151 (2)	1.0410 (8)	0.2510 (7)	0.057 (2)	0.5
H5	0.230184	1.052526	0.311120	0.068*	0.5
C6	0.053 (2)	0.9385 (8)	0.2135 (6)	0.046 (2)	0.5
H6	0.087011	0.878548	0.244745	0.055*	0.5
C7	−0.2107 (8)	0.8138 (3)	0.0956 (3)	0.0471 (9)	
C8A	−0.477 (4)	0.804 (2)	0.0421 (15)	0.062 (5)	0.5
H8A	−0.457921	0.763747	−0.017873	0.075*	0.5
H8B	−0.540448	0.877297	0.036156	0.075*	0.5
H8C	−0.606766	0.765985	0.072809	0.075*	0.5
C8	−0.405 (4)	0.7988 (19)	0.0111 (15)	0.067 (6)	0.5
H8D	−0.329965	0.748035	−0.036166	0.081*	0.5
H8E	−0.430501	0.869171	−0.009852	0.081*	0.5
H8F	−0.577752	0.769499	0.024940	0.081*	0.5
C9	−0.0943 (7)	0.5445 (3)	0.1251 (2)	0.0363 (7)	
C10	0.2629 (7)	0.4508 (3)	0.2385 (2)	0.0406 (8)	
H10A	0.105264	0.412724	0.257139	0.049*	
H10B	0.330537	0.404971	0.185987	0.049*	
C11	0.4822 (7)	0.4701 (3)	0.3158 (2)	0.0421 (8)	
H11A	0.637148	0.509765	0.297023	0.051*	
H11B	0.412573	0.515802	0.368017	0.051*	
C12	0.5748 (7)	0.3632 (3)	0.3436 (2)	0.0433 (8)	
H12A	0.651622	0.319515	0.291733	0.052*	
H12B	0.416427	0.321934	0.358284	0.052*	
C13	0.7831 (8)	0.3763 (3)	0.4239 (2)	0.0458 (8)	
H13A	0.941383	0.417864	0.409574	0.055*	
H13B	0.706096	0.419128	0.476121	0.055*	
C14	0.8745 (8)	0.2683 (3)	0.4499 (3)	0.0479 (9)	
H14A	0.714985	0.226115	0.462490	0.057*	
H14B	0.954852	0.226428	0.398009	0.057*	
C15	1.0773 (8)	0.2790 (4)	0.5313 (3)	0.0520 (9)	
H15A	1.237728	0.320556	0.518707	0.062*	
H15B	0.997681	0.321188	0.583283	0.062*	
C16	1.1647 (10)	0.1709 (4)	0.5568 (3)	0.0652 (12)	
H16A	1.244326	0.128450	0.504959	0.078*	
H16B	1.004888	0.129348	0.569939	0.078*	
C17	1.3697 (11)	0.1840 (5)	0.6387 (4)	0.0820 (16)	
H17A	1.429776	0.112155	0.649602	0.098*	
H17B	1.286255	0.219771	0.691591	0.098*	
H17C	1.524302	0.228373	0.627259	0.098*	

### S-n-Octyl 3-(1-phenylethylidene)dithiocarbazate (HL) Atomic displacement parameters (Å^2^)

**Table:**

	*U*^11^	*U*^22^	*U*^33^	*U*^12^	*U*^13^	*U*^23^
N1	0.0423 (16)	0.0350 (14)	0.0419 (16)	−0.0025 (12)	−0.0012 (13)	0.0052 (12)
N2	0.0427 (16)	0.0373 (15)	0.0416 (16)	−0.0035 (12)	−0.0029 (14)	0.0062 (13)
S1	0.0482 (5)	0.0357 (4)	0.0442 (5)	−0.0042 (4)	−0.0046 (4)	0.0053 (4)
S2	0.0441 (5)	0.0387 (5)	0.0449 (5)	−0.0039 (4)	−0.0062 (4)	0.0064 (4)
C1	0.0404 (18)	0.0364 (17)	0.049 (2)	0.0006 (14)	−0.0064 (16)	0.0067 (15)
C2A	0.053 (5)	0.042 (4)	0.054 (5)	0.006 (4)	−0.011 (4)	0.003 (4)
C3A	0.073 (7)	0.032 (4)	0.061 (6)	0.005 (4)	−0.009 (5)	0.003 (4)
C2	0.043 (4)	0.043 (4)	0.048 (4)	0.004 (3)	−0.012 (4)	0.001 (4)
C3	0.045 (5)	0.037 (4)	0.065 (6)	−0.002 (4)	−0.005 (4)	0.017 (4)
C4	0.075 (3)	0.038 (2)	0.067 (3)	−0.0090 (19)	−0.016 (2)	0.0005 (19)
C5A	0.060 (6)	0.050 (5)	0.064 (6)	−0.004 (5)	−0.020 (5)	0.002 (5)
C6A	0.045 (5)	0.044 (4)	0.058 (6)	−0.007 (4)	−0.008 (4)	0.011 (4)
C5	0.061 (6)	0.053 (5)	0.050 (5)	0.000 (5)	−0.013 (4)	−0.004 (4)
C6	0.053 (6)	0.039 (4)	0.045 (5)	−0.002 (4)	−0.007 (4)	0.006 (4)
C7	0.0436 (19)	0.0381 (18)	0.057 (2)	−0.0040 (15)	−0.0158 (17)	0.0091 (16)
C8A	0.046 (8)	0.047 (6)	0.089 (15)	−0.002 (5)	−0.026 (7)	0.007 (9)
C8	0.075 (14)	0.035 (5)	0.083 (13)	−0.001 (8)	−0.039 (9)	0.006 (7)
C9	0.0375 (17)	0.0376 (17)	0.0341 (16)	−0.0016 (13)	0.0065 (14)	0.0047 (13)
C10	0.0409 (18)	0.0404 (18)	0.0407 (18)	0.0006 (14)	0.0001 (15)	0.0080 (15)
C11	0.0419 (19)	0.0436 (19)	0.0394 (18)	−0.0007 (15)	−0.0022 (15)	0.0045 (15)
C12	0.0411 (19)	0.047 (2)	0.0403 (19)	−0.0044 (15)	−0.0051 (15)	0.0073 (15)
C13	0.046 (2)	0.052 (2)	0.0380 (19)	−0.0030 (16)	−0.0043 (16)	0.0056 (16)
C14	0.0417 (19)	0.057 (2)	0.044 (2)	−0.0016 (16)	−0.0046 (16)	0.0084 (17)
C15	0.046 (2)	0.065 (3)	0.045 (2)	0.0032 (18)	−0.0029 (17)	0.0120 (19)
C16	0.059 (3)	0.076 (3)	0.065 (3)	0.008 (2)	−0.004 (2)	0.027 (2)
C17	0.069 (3)	0.114 (5)	0.067 (3)	0.018 (3)	−0.010 (3)	0.034 (3)

### S-n-Octyl 3-(1-phenylethylidene)dithiocarbazate (HL) Geometric parameters (Å, º)

**Table:**

N1—C7	1.284 (4)	C8A—H8A	0.9800
N1—N2	1.377 (4)	C8A—H8B	0.9800
N2—C9	1.340 (4)	C8A—H8C	0.9800
N2—H2N	0.87 (4)	C8—H8D	0.9800
S1—C9	1.669 (3)	C8—H8E	0.9800
S2—C9	1.750 (3)	C8—H8F	0.9800
S2—C10	1810 (3)	C10—C11	1.526 (5)
C1—C6	1.342 (10)	C10—H10A	0.9900
C1—C2A	1.399 (9)	C10—H10B	0.9900
C1—C6A	1.407 (10)	C11—C12	1.521 (5)
C1—C2	1.438 (9)	C11—H11A	0.9900
C1—C7	1.484 (5)	C11—H11B	0.9900
C2A—C3A	1.387 (12)	C12—C13	1.523 (5)
C2A—H2A	0.9500	C12—H12A	0.9900
C3A—C4	1.323 (11)	C12—H12B	0.9900
C3A—H3A	0.9500	C13—C14	1.521 (5)
C2—C3	1.390 (12)	C13—H13A	0.9900
C2—H2	0.9500	C13—H13B	0.9900
C3—C4	1.382 (10)	C14—C15	1.519 (5)
C3—H3	0.9500	C14—H14A	0.9900
C4—C5	1.377 (11)	C14—H14B	0.9900
C4—C5A	1.413 (11)	C15—C16	1.513 (6)
C4—H4	0.9500	C15—H15A	0.9900
C5A—C6A	1.390 (13)	C15—H15B	0.9900
C5A—H5A	0.9500	C16—C17	1.530 (6)
C6A—H6A	0.9500	C16—H16A	0.9900
C5—C6	1.380 (13)	C16—H16B	0.9900
C5—H5	0.9500	C17—H17A	0.9800
C6—H6	0.9500	C17—H17B	0.9800
C7—C8A	1.50 (2)	C17—H17C	0.9800
C7—C8	1.53 (2)		
			
C7—N1—N2	118.4 (3)	H8D—C8—H8F	109.5
C9—N2—N1	118.4 (3)	H8E—C8—H8F	109.5
C9—N2—H2N	117 (2)	N2—C9—S1	120.8 (3)
N1—N2—H2N	124 (2)	N2—C9—S2	113.6 (2)
C9—S2—C10	102.08 (16)	S1—C9—S2	125.6 (2)
C2A—C1—C6A	117.8 (6)	C11—C10—S2	108.4 (2)
C6—C1—C2	119.0 (6)	C11—C10—H10A	110.0
C6—C1—C7	120.9 (5)	S2—C10—H10A	110.0
C2A—C1—C7	121.9 (4)	C11—C10—H10B	110.0
C6A—C1—C7	120.1 (5)	S2—C10—H10B	110.0
C2—C1—C7	119.9 (4)	H10A—C10—H10B	108.4
C3A—C2A—C1	120.5 (8)	C12—C11—C10	111.2 (3)
C3A—C2A—H2A	119.8	C12—C11—H11A	109.4
C1—C2A—H2A	119.8	C10—C11—H11A	109.4
C4—C3A—C2A	121.0 (8)	C12—C11—H11B	109.4
C4—C3A—H3A	119.5	C10—C11—H11B	109.4
C2A—C3A—H3A	119.5	H11A—C11—H11B	108.0
C3—C2—C1	119.1 (7)	C11—C12—C13	114.1 (3)
C3—C2—H2	120.4	C11—C12—H12A	108.7
C1—C2—H2	120.4	C13—C12—H12A	108.7
C4—C3—C2	119.9 (8)	C11—C12—H12B	108.7
C4—C3—H3	120.1	C13—C12—H12B	108.7
C2—C3—H3	120.1	H12A—C12—H12B	107.6
C5—C4—C3	119.4 (6)	C14—C13—C12	113.2 (3)
C3A—C4—C5A	121.0 (6)	C14—C13—H13A	108.9
C5—C4—H4	120.3	C12—C13—H13A	108.9
C3—C4—H4	120.3	C14—C13—H13B	108.9
C6A—C5A—C4	118.9 (8)	C12—C13—H13B	108.9
C6A—C5A—H5A	120.6	H13A—C13—H13B	107.8
C4—C5A—H5A	120.6	C15—C14—C13	114.3 (3)
C5A—C6A—C1	120.2 (9)	C15—C14—H14A	108.7
C5A—C6A—H6A	119.9	C13—C14—H14A	108.7
C1—C6A—H6A	119.9	C15—C14—H14B	108.7
C4—C5—C6	121.0 (8)	C13—C14—H14B	108.7
C4—C5—H5	119.5	H14A—C14—H14B	107.6
C6—C5—H5	119.5	C16—C15—C14	113.7 (4)
C1—C6—C5	120.8 (8)	C16—C15—H15A	108.8
C1—C6—H6	119.6	C14—C15—H15A	108.8
C5—C6—H6	119.6	C16—C15—H15B	108.8
N1—C7—C1	116.1 (3)	C14—C15—H15B	108.8
N1—C7—C8A	122.2 (10)	H15A—C15—H15B	107.7
C1—C7—C8A	120.2 (10)	C15—C16—C17	112.6 (4)
N1—C7—C8	121.7 (9)	C15—C16—H16A	109.1
C1—C7—C8	120.9 (9)	C17—C16—H16A	109.1
C7—C8A—H8A	109.5	C15—C16—H16B	109.1
C7—C8A—H8B	109.5	C17—C16—H16B	109.1
H8A—C8A—H8B	109.5	H16A—C16—H16B	107.8
C7—C8A—H8C	109.5	C16—C17—H17A	109.5
H8A—C8A—H8C	109.5	C16—C17—H17B	109.5
H8B—C8A—H8C	109.5	H17A—C17—H17B	109.5
C7—C8—H8D	109.5	C16—C17—H17C	109.5
C7—C8—H8E	109.5	H17A—C17—H17C	109.5
H8D—C8—H8E	109.5	H17B—C17—H17C	109.5
C7—C8—H8F	109.5		
			
C7—N1—N2—C9	178.7 (3)	C6—C1—C7—N1	−21.6 (7)
C6A—C1—C2A—C3A	9.7 (13)	C2A—C1—C7—N1	−159.1 (6)
C7—C1—C2A—C3A	−175.8 (8)	C6A—C1—C7—N1	15.2 (7)
C1—C2A—C3A—C4	−7.6 (16)	C2—C1—C7—N1	153.7 (5)
C6—C1—C2—C3	−7.5 (12)	C2A—C1—C7—C8A	7.0 (11)
C7—C1—C2—C3	177.2 (7)	C6A—C1—C7—C8A	−178.7 (10)
C1—C2—C3—C4	6.1 (13)	C6—C1—C7—C8	171.6 (10)
C2A—C3A—C4—C5A	4.2 (15)	C2—C1—C7—C8	−13.2 (11)
C2—C3—C4—C5	−5.9 (13)	N1—N2—C9—S1	−177.0 (2)
C3A—C4—C5A—C6A	−3.3 (15)	N1—N2—C9—S2	3.0 (4)
C4—C5A—C6A—C1	5.7 (16)	C10—S2—C9—N2	177.4 (3)
C2A—C1—C6A—C5A	−8.9 (13)	C10—S2—C9—S1	−2.6 (3)
C7—C1—C6A—C5A	176.6 (8)	C9—S2—C10—C11	−176.7 (2)
C3—C4—C5—C6	7.0 (14)	S2—C10—C11—C12	−179.2 (3)
C2—C1—C6—C5	8.6 (13)	C10—C11—C12—C13	−176.8 (3)
C7—C1—C6—C5	−176.0 (8)	C11—C12—C13—C14	−179.5 (3)
C4—C5—C6—C1	−8.6 (16)	C12—C13—C14—C15	−178.6 (3)
N2—N1—C7—C1	179.8 (3)	C13—C14—C15—C16	179.6 (4)
N2—N1—C7—C8A	14.0 (10)	C14—C15—C16—C17	179.8 (4)
N2—N1—C7—C8	−13.4 (10)		

### Bis[S-n-octyl 3-(1-phenylethylidene)dithiocarbazato]nickel(II) (NiL2) Crystal data

**Table:**

[Ni(C_17_H_25_N_2_S_2_)_2_]	*F*(000) = 1496
*M**_r_* = 701.73	*D*_x_ = 1.264 Mg m^−^^3^
Monoclinic, *P*2_1_/*n*	Mo *K*α radiation, λ = 0.71075 Å
*a* = 13.6399 (3) Å	Cell parameters from 26150 reflections
*b* = 17.6532 (5) Å	θ = 1.8–27.5°
*c* = 16.7596 (3) Å	µ = 0.78 mm^−^^1^
β = 114.000 (8)°	*T* = 173 K
*V* = 3686.6 (3) Å^3^	Needle, green
*Z* = 4	0.27 × 0.09 × 0.03 mm

### Bis[S-n-octyl 3-(1-phenylethylidene)dithiocarbazato]nickel(II) (NiL2) Data collection

**Table:**

Rigaku R-AXIS RAPID diffractometer	6674 reflections with *I* > 2σ(*I*)
Detector resolution: 10.000 pixels mm^-1^	*R*_int_ = 0.038
ω scans	θ_max_ = 27.5°, θ_min_ = 1.8°
Absorption correction: multi-scan (ABSCOR; Higashi, 1995)	*h* = −17→17
*T*_min_ = 0.737, *T*_max_ = 0.977	*k* = −22→22
35807 measured reflections	*l* = −20→21
8419 independent reflections	

### Bis[S-n-octyl 3-(1-phenylethylidene)dithiocarbazato]nickel(II) (NiL2) Refinement

**Table:**

Refinement on *F*^2^	0 restraints
Least-squares matrix: full	Hydrogen site location: inferred from neighbouring sites
*R*[*F*^2^ > 2σ(*F*^2^)] = 0.042	H-atom parameters constrained
*wR*(*F*^2^) = 0.096	*w* = 1/[σ^2^(*F*_o_^2^) + (0.0435*P*)^2^ + 1.4889*P*] where *P* = (*F*_o_^2^ + 2*F*_c_^2^)/3
*S* = 1.04	(Δ/σ)_max_ = 0.001
8419 reflections	Δρ_max_ = 0.60 e Å^−^^3^
392 parameters	Δρ_min_ = −0.25 e Å^−^^3^

### Bis[S-n-octyl 3-(1-phenylethylidene)dithiocarbazato]nickel(II) (NiL2) Special details

**Table:**

Geometry. All esds (except the esd in the dihedral angle between two l.s. planes) are estimated using the full covariance matrix. The cell esds are taken into account individually in the estimation of esds in distances, angles and torsion angles; correlations between esds in cell parameters are only used when they are defined by crystal symmetry. An approximate (isotropic) treatment of cell esds is used for estimating esds involving l.s. planes.

### Bis[S-n-octyl 3-(1-phenylethylidene)dithiocarbazato]nickel(II) (NiL2) Fractional atomic coordinates and isotropic or equivalent isotropic displacement parameters (Å^2^)

**Table:**

	*x*	*y*	*z*	*U*_iso_*/*U*_eq_	
Ni1	0.74039 (2)	0.25330 (2)	0.25385 (2)	0.03168 (8)	
S1	0.84963 (5)	0.16206 (3)	0.31516 (4)	0.04322 (14)	
S2	1.08344 (5)	0.17784 (3)	0.40403 (4)	0.05019 (16)	
S3	0.61639 (5)	0.17236 (3)	0.18452 (4)	0.04295 (14)	
S4	0.38828 (5)	0.20819 (3)	0.08597 (4)	0.04600 (14)	
N1	0.84293 (13)	0.31828 (8)	0.34158 (10)	0.0311 (3)	
N2	0.95110 (13)	0.29419 (9)	0.37651 (10)	0.0357 (4)	
N3	0.65212 (13)	0.32728 (8)	0.17072 (10)	0.0301 (3)	
N4	0.54030 (13)	0.31270 (9)	0.13143 (10)	0.0345 (4)	
C1	0.71951 (16)	0.41247 (10)	0.34871 (12)	0.0313 (4)	
C2	0.63589 (16)	0.36389 (11)	0.34242 (12)	0.0353 (4)	
H2	0.649382	0.311221	0.352804	0.042*	
C3	0.53335 (17)	0.39187 (12)	0.32114 (14)	0.0423 (5)	
H3	0.476887	0.358265	0.316505	0.051*	
C4	0.51303 (18)	0.46818 (13)	0.30670 (15)	0.0455 (5)	
H4	0.442648	0.487096	0.292229	0.055*	
C5	0.59488 (19)	0.51736 (12)	0.31323 (15)	0.0456 (5)	
H5	0.580546	0.569943	0.302850	0.055*	
C6	0.69736 (17)	0.49012 (10)	0.33480 (13)	0.0369 (4)	
H6	0.753509	0.524340	0.340292	0.044*	
C7	0.82927 (16)	0.38357 (10)	0.37186 (12)	0.0316 (4)	
C8	0.92187 (17)	0.43050 (11)	0.43057 (14)	0.0405 (5)	
H8A	0.967649	0.400021	0.480793	0.049*	
H8B	0.894927	0.474549	0.451068	0.049*	
H8C	0.963667	0.447671	0.398296	0.049*	
C9	0.95825 (17)	0.22217 (11)	0.36603 (13)	0.0367 (4)	
C10	1.17362 (18)	0.25061 (14)	0.46973 (15)	0.0509 (6)	
H10A	1.140263	0.275184	0.505639	0.061*	
H10B	1.240558	0.225943	0.510265	0.061*	
C11	1.20302 (19)	0.31163 (14)	0.41984 (15)	0.0513 (6)	
H11A	1.138161	0.341163	0.384373	0.062*	
H11B	1.230706	0.287810	0.379651	0.062*	
C12	1.2880 (2)	0.36459 (17)	0.48235 (18)	0.0684 (8)	
H12A	1.261218	0.385392	0.524572	0.082*	
H12B	1.353331	0.334666	0.515807	0.082*	
C13	1.3188 (2)	0.43029 (17)	0.4382 (2)	0.0740 (8)	
H13A	1.389438	0.450271	0.478590	0.089*	
H13B	1.326489	0.411161	0.385544	0.089*	
C14	1.2390 (2)	0.49362 (15)	0.41222 (17)	0.0594 (7)	
H14A	1.239582	0.517821	0.465697	0.071*	
H14B	1.166457	0.472263	0.379554	0.071*	
C15	1.2602 (2)	0.55481 (15)	0.35546 (16)	0.0587 (7)	
H15A	1.269751	0.529466	0.306412	0.070*	
H15B	1.195840	0.587481	0.330127	0.070*	
C16	1.3558 (2)	0.60421 (18)	0.40216 (18)	0.0683 (7)	
H16A	1.420947	0.572151	0.424799	0.082*	
H16B	1.348212	0.627938	0.452884	0.082*	
C17	1.3710 (3)	0.66657 (19)	0.3449 (2)	0.0846 (9)	
H17A	1.432010	0.698486	0.380235	0.102*	
H17B	1.305876	0.697621	0.320527	0.102*	
H17C	1.384727	0.643577	0.297226	0.102*	
C18	0.79429 (16)	0.40675 (10)	0.17028 (12)	0.0311 (4)	
C19	0.86459 (16)	0.34878 (11)	0.17231 (12)	0.0341 (4)	
H19	0.838889	0.298260	0.159744	0.041*	
C20	0.97162 (17)	0.36419 (13)	0.19250 (13)	0.0414 (5)	
H20	1.019032	0.324289	0.193909	0.050*	
C21	1.00957 (18)	0.43770 (14)	0.21063 (14)	0.0455 (5)	
H21	1.082960	0.448151	0.224205	0.055*	
C22	0.94112 (19)	0.49591 (13)	0.20904 (14)	0.0457 (5)	
H22	0.967704	0.546150	0.222558	0.055*	
C23	0.83347 (17)	0.48093 (11)	0.18769 (13)	0.0372 (4)	
H23	0.786094	0.521277	0.184876	0.045*	
C24	0.67924 (16)	0.39092 (10)	0.14602 (12)	0.0309 (4)	
C25	0.59705 (17)	0.44680 (11)	0.09090 (14)	0.0386 (5)	
H25A	0.553452	0.423904	0.034202	0.046*	
H25B	0.633169	0.491993	0.082072	0.046*	
H25C	0.550715	0.461094	0.120355	0.046*	
C26	0.52026 (17)	0.24162 (11)	0.13485 (13)	0.0358 (4)	
C27	0.31613 (18)	0.29143 (12)	0.02909 (14)	0.0436 (5)	
H27A	0.358150	0.316283	0.000245	0.052*	
H27B	0.246964	0.275151	−0.017279	0.052*	
C28	0.29357 (18)	0.34919 (12)	0.08641 (14)	0.0436 (5)	
H28A	0.245687	0.326397	0.111130	0.052*	
H28B	0.361711	0.363080	0.135586	0.052*	
C29	0.24138 (18)	0.41999 (12)	0.03573 (14)	0.0437 (5)	
H29A	0.290487	0.443173	0.012497	0.052*	
H29B	0.174836	0.405413	−0.014688	0.052*	
C30	0.2140 (2)	0.47840 (13)	0.08941 (15)	0.0488 (5)	
H30A	0.170690	0.453715	0.117230	0.059*	
H30B	0.281430	0.496272	0.136649	0.059*	
C31	0.15295 (19)	0.54626 (12)	0.03832 (15)	0.0456 (5)	
H31A	0.197589	0.572328	0.012675	0.055*	
H31B	0.087024	0.528287	−0.010493	0.055*	
C32	0.1218 (2)	0.60272 (13)	0.09154 (15)	0.0516 (6)	
H32A	0.083050	0.575616	0.121678	0.062*	
H32B	0.187996	0.624210	0.136973	0.062*	
C33	0.0526 (2)	0.66669 (14)	0.03960 (17)	0.0542 (6)	
H33A	−0.013476	0.645405	−0.006231	0.065*	
H33B	0.091524	0.694443	0.010095	0.065*	
C34	0.0215 (2)	0.72192 (17)	0.0943 (2)	0.0741 (8)	
H34A	−0.018940	0.695217	0.122478	0.089*	
H34B	−0.023206	0.762201	0.056803	0.089*	
H34C	0.086370	0.744135	0.139102	0.089*	

### Bis[S-n-octyl 3-(1-phenylethylidene)dithiocarbazato]nickel(II) (NiL2) Atomic displacement parameters (Å^2^)

**Table:**

	*U*^11^	*U*^22^	*U*^33^	*U*^12^	*U*^13^	*U*^23^
Ni1	0.04200 (15)	0.01830 (12)	0.03551 (14)	0.00075 (10)	0.01657 (11)	0.00046 (9)
S1	0.0548 (3)	0.0224 (2)	0.0521 (3)	0.0068 (2)	0.0213 (3)	0.0038 (2)
S2	0.0516 (4)	0.0419 (3)	0.0579 (4)	0.0196 (3)	0.0231 (3)	0.0148 (3)
S3	0.0504 (3)	0.0206 (2)	0.0551 (3)	−0.0034 (2)	0.0186 (3)	−0.0014 (2)
S4	0.0446 (3)	0.0315 (3)	0.0624 (4)	−0.0102 (2)	0.0223 (3)	−0.0078 (2)
N1	0.0366 (9)	0.0239 (7)	0.0340 (9)	0.0022 (6)	0.0156 (7)	0.0030 (6)
N2	0.0384 (9)	0.0319 (9)	0.0361 (9)	0.0075 (7)	0.0142 (7)	0.0052 (7)
N3	0.0372 (9)	0.0213 (7)	0.0337 (8)	−0.0018 (6)	0.0164 (7)	−0.0028 (6)
N4	0.0381 (9)	0.0254 (8)	0.0395 (9)	−0.0036 (7)	0.0155 (7)	−0.0018 (6)
C1	0.0429 (11)	0.0253 (9)	0.0295 (10)	0.0009 (8)	0.0187 (8)	−0.0022 (7)
C2	0.0466 (12)	0.0269 (9)	0.0363 (11)	−0.0003 (8)	0.0207 (9)	0.0015 (8)
C3	0.0435 (12)	0.0420 (12)	0.0464 (12)	−0.0015 (9)	0.0235 (10)	0.0030 (9)
C4	0.0449 (13)	0.0489 (13)	0.0487 (13)	0.0112 (10)	0.0252 (10)	0.0052 (10)
C5	0.0617 (15)	0.0316 (11)	0.0521 (13)	0.0112 (10)	0.0318 (11)	0.0041 (9)
C6	0.0501 (12)	0.0250 (9)	0.0437 (12)	−0.0003 (8)	0.0274 (10)	−0.0025 (8)
C7	0.0400 (11)	0.0256 (9)	0.0318 (10)	−0.0012 (8)	0.0174 (8)	0.0012 (7)
C8	0.0437 (12)	0.0318 (10)	0.0455 (12)	−0.0057 (9)	0.0175 (10)	−0.0067 (9)
C9	0.0453 (12)	0.0318 (10)	0.0353 (11)	0.0084 (9)	0.0189 (9)	0.0084 (8)
C10	0.0429 (13)	0.0663 (16)	0.0394 (12)	0.0159 (11)	0.0127 (10)	0.0135 (11)
C11	0.0460 (14)	0.0620 (15)	0.0448 (13)	0.0089 (11)	0.0173 (11)	0.0091 (11)
C12	0.0576 (17)	0.0686 (19)	0.0663 (17)	0.0020 (14)	0.0123 (14)	0.0031 (14)
C13	0.0522 (17)	0.072 (2)	0.093 (2)	0.0032 (14)	0.0239 (15)	0.0015 (16)
C14	0.0557 (16)	0.0690 (18)	0.0522 (15)	0.0069 (13)	0.0204 (12)	−0.0060 (12)
C15	0.0654 (17)	0.0623 (16)	0.0492 (14)	0.0117 (13)	0.0242 (13)	−0.0067 (12)
C16	0.0590 (17)	0.083 (2)	0.0584 (17)	0.0056 (15)	0.0191 (13)	−0.0046 (14)
C17	0.078 (2)	0.081 (2)	0.095 (2)	−0.0064 (18)	0.0353 (19)	−0.0062 (18)
C18	0.0414 (11)	0.0269 (9)	0.0277 (9)	−0.0020 (8)	0.0170 (8)	0.0014 (7)
C19	0.0444 (12)	0.0314 (10)	0.0276 (10)	0.0006 (8)	0.0159 (8)	0.0001 (7)
C20	0.0425 (12)	0.0505 (13)	0.0335 (11)	0.0057 (10)	0.0177 (9)	0.0017 (9)
C21	0.0420 (12)	0.0617 (15)	0.0364 (12)	−0.0125 (11)	0.0196 (10)	−0.0019 (10)
C22	0.0565 (14)	0.0423 (12)	0.0443 (13)	−0.0175 (10)	0.0268 (11)	−0.0056 (9)
C23	0.0487 (12)	0.0283 (10)	0.0413 (11)	−0.0049 (9)	0.0252 (10)	−0.0011 (8)
C24	0.0405 (11)	0.0219 (9)	0.0324 (10)	−0.0012 (8)	0.0168 (8)	−0.0029 (7)
C25	0.0452 (12)	0.0241 (9)	0.0453 (12)	0.0017 (8)	0.0172 (10)	0.0036 (8)
C26	0.0444 (12)	0.0273 (10)	0.0397 (11)	−0.0045 (8)	0.0213 (9)	−0.0047 (8)
C27	0.0408 (12)	0.0426 (12)	0.0443 (12)	−0.0029 (10)	0.0140 (10)	−0.0072 (9)
C28	0.0453 (13)	0.0436 (12)	0.0464 (13)	0.0002 (10)	0.0231 (10)	−0.0001 (9)
C29	0.0476 (13)	0.0458 (13)	0.0416 (12)	0.0006 (10)	0.0222 (10)	0.0012 (9)
C30	0.0579 (15)	0.0465 (13)	0.0435 (13)	0.0084 (11)	0.0221 (11)	0.0030 (10)
C31	0.0491 (13)	0.0419 (12)	0.0484 (13)	0.0033 (10)	0.0224 (11)	−0.0014 (10)
C32	0.0588 (15)	0.0465 (13)	0.0504 (14)	0.0055 (11)	0.0231 (11)	−0.0037 (10)
C33	0.0506 (15)	0.0472 (14)	0.0673 (16)	0.0037 (11)	0.0267 (12)	−0.0052 (11)
C34	0.072 (2)	0.0666 (18)	0.084 (2)	0.0144 (15)	0.0318 (16)	−0.0169 (16)

### Bis[S-n-octyl 3-(1-phenylethylidene)dithiocarbazato]nickel(II) (NiL2) Geometric parameters (Å, º)

**Table:**

Ni1—N3	1.9318 (15)	C15—H15A	0.9900
Ni1—N1	1.9392 (16)	C15—H15B	0.9900
Ni1—S1	2.1506 (6)	C16—C17	1.529 (4)
Ni1—S3	2.1573 (6)	C16—H16A	0.9900
S1—C9	1.738 (2)	C16—H16B	0.9900
S2—C9	1.746 (2)	C17—H17A	0.9800
S2—C10	1.809 (3)	C17—H17B	0.9800
S3—C26	1.738 (2)	C17—H17C	0.9800
S4—C26	1.749 (2)	C18—C19	1.393 (3)
S4—C27	1.809 (2)	C18—C23	1.400 (3)
N1—C7	1.303 (2)	C18—C24	1.479 (3)
N1—N2	1.414 (2)	C19—C20	1.385 (3)
N2—C9	1.293 (3)	C19—H19	0.9500
N3—C24	1.302 (2)	C20—C21	1.384 (3)
N3—N4	1.417 (2)	C20—H20	0.9500
N4—C26	1.290 (2)	C21—C22	1.381 (3)
C1—C2	1.396 (3)	C21—H21	0.9500
C1—C6	1.403 (3)	C22—C23	1.388 (3)
C1—C7	1.476 (3)	C22—H22	0.9500
C2—C3	1.386 (3)	C23—H23	0.9500
C2—H2	0.9500	C24—C25	1.497 (3)
C3—C4	1.377 (3)	C25—H25A	0.9800
C3—H3	0.9500	C25—H25B	0.9800
C4—C5	1.383 (3)	C25—H25C	0.9800
C4—H4	0.9500	C27—C28	1.516 (3)
C5—C6	1.380 (3)	C27—H27A	0.9900
C5—H5	0.9500	C27—H27B	0.9900
C6—H6	0.9500	C28—C29	1.516 (3)
C7—C8	1.496 (3)	C28—H28A	0.9900
C8—H8A	0.9800	C28—H28B	0.9900
C8—H8B	0.9800	C29—C30	1.512 (3)
C8—H8C	0.9800	C29—H29A	0.9900
C10—C11	1.514 (3)	C29—H29B	0.9900
C10—H10A	0.9900	C30—C31	1.510 (3)
C10—H10B	0.9900	C30—H30A	0.9900
C11—C12	1.526 (4)	C30—H30B	0.9900
C11—H11A	0.9900	C31—C32	1.509 (3)
C11—H11B	0.9900	C31—H31A	0.9900
C12—C13	1.523 (4)	C31—H31B	0.9900
C12—H12A	0.9900	C32—C33	1.502 (3)
C12—H12B	0.9900	C32—H32A	0.9900
C13—C14	1.496 (4)	C32—H32B	0.9900
C13—H13A	0.9900	C33—C34	1.513 (3)
C13—H13B	0.9900	C33—H33A	0.9900
C14—C15	1.542 (4)	C33—H33B	0.9900
C14—H14A	0.9900	C34—H34A	0.9800
C14—H14B	0.9900	C34—H34B	0.9800
C15—C16	1.498 (4)	C34—H34C	0.9800
			
N3—Ni1—N1	101.11 (7)	C15—C16—H16B	108.8
N3—Ni1—S1	163.41 (5)	C17—C16—H16B	108.8
N1—Ni1—S1	86.26 (5)	H16A—C16—H16B	107.7
N3—Ni1—S3	86.40 (5)	C16—C17—H17A	109.5
N1—Ni1—S3	164.97 (5)	C16—C17—H17B	109.5
S1—Ni1—S3	90.02 (2)	H17A—C17—H17B	109.5
C9—S1—Ni1	93.66 (7)	C16—C17—H17C	109.5
C9—S2—C10	103.05 (10)	H17A—C17—H17C	109.5
C26—S3—Ni1	93.75 (7)	H17B—C17—H17C	109.5
C26—S4—C27	102.03 (10)	C19—C18—C23	118.78 (18)
C7—N1—N2	113.29 (16)	C19—C18—C24	120.72 (17)
C7—N1—Ni1	130.19 (14)	C23—C18—C24	120.44 (17)
N2—N1—Ni1	116.46 (11)	C20—C19—C18	120.55 (19)
C9—N2—N1	111.31 (16)	C20—C19—H19	119.7
C24—N3—N4	113.59 (15)	C18—C19—H19	119.7
C24—N3—Ni1	129.67 (14)	C21—C20—C19	120.0 (2)
N4—N3—Ni1	116.71 (11)	C21—C20—H20	120.0
C26—N4—N3	111.30 (16)	C19—C20—H20	120.0
C2—C1—C6	118.27 (18)	C22—C21—C20	120.2 (2)
C2—C1—C7	121.16 (16)	C22—C21—H21	119.9
C6—C1—C7	120.55 (18)	C20—C21—H21	119.9
C3—C2—C1	120.55 (18)	C21—C22—C23	119.9 (2)
C3—C2—H2	119.7	C21—C22—H22	120.0
C1—C2—H2	119.7	C23—C22—H22	120.0
C4—C3—C2	120.3 (2)	C22—C23—C18	120.4 (2)
C4—C3—H3	119.9	C22—C23—H23	119.8
C2—C3—H3	119.9	C18—C23—H23	119.8
C3—C4—C5	120.1 (2)	N3—C24—C18	118.85 (17)
C3—C4—H4	119.9	N3—C24—C25	121.78 (18)
C5—C4—H4	119.9	C18—C24—C25	119.33 (16)
C6—C5—C4	120.1 (2)	C24—C25—H25A	109.5
C6—C5—H5	120.0	C24—C25—H25B	109.5
C4—C5—H5	120.0	H25A—C25—H25B	109.5
C5—C6—C1	120.72 (19)	C24—C25—H25C	109.5
C5—C6—H6	119.6	H25A—C25—H25C	109.5
C1—C6—H6	119.6	H25B—C25—H25C	109.5
N1—C7—C1	119.58 (17)	N4—C26—S3	124.99 (16)
N1—C7—C8	121.99 (18)	N4—C26—S4	120.22 (16)
C1—C7—C8	118.42 (16)	S3—C26—S4	114.79 (11)
C7—C8—H8A	109.5	C28—C27—S4	114.80 (15)
C7—C8—H8B	109.5	C28—C27—H27A	108.6
H8A—C8—H8B	109.5	S4—C27—H27A	108.6
C7—C8—H8C	109.5	C28—C27—H27B	108.6
H8A—C8—H8C	109.5	S4—C27—H27B	108.6
H8B—C8—H8C	109.5	H27A—C27—H27B	107.5
N2—C9—S1	124.81 (16)	C29—C28—C27	111.69 (18)
N2—C9—S2	120.52 (16)	C29—C28—H28A	109.3
S1—C9—S2	114.67 (11)	C27—C28—H28A	109.3
C11—C10—S2	115.86 (17)	C29—C28—H28B	109.3
C11—C10—H10A	108.3	C27—C28—H28B	109.3
S2—C10—H10A	108.3	H28A—C28—H28B	107.9
C11—C10—H10B	108.3	C30—C29—C28	113.72 (18)
S2—C10—H10B	108.3	C30—C29—H29A	108.8
H10A—C10—H10B	107.4	C28—C29—H29A	108.8
C10—C11—C12	110.8 (2)	C30—C29—H29B	108.8
C10—C11—H11A	109.5	C28—C29—H29B	108.8
C12—C11—H11A	109.5	H29A—C29—H29B	107.7
C10—C11—H11B	109.5	C31—C30—C29	114.60 (18)
C12—C11—H11B	109.5	C31—C30—H30A	108.6
H11A—C11—H11B	108.1	C29—C30—H30A	108.6
C13—C12—C11	114.6 (2)	C31—C30—H30B	108.6
C13—C12—H12A	108.6	C29—C30—H30B	108.6
C11—C12—H12A	108.6	H30A—C30—H30B	107.6
C13—C12—H12B	108.6	C32—C31—C30	114.30 (19)
C11—C12—H12B	108.6	C32—C31—H31A	108.7
H12A—C12—H12B	107.6	C30—C31—H31A	108.7
C14—C13—C12	113.5 (2)	C32—C31—H31B	108.7
C14—C13—H13A	108.9	C30—C31—H31B	108.7
C12—C13—H13A	108.9	H31A—C31—H31B	107.6
C14—C13—H13B	108.9	C33—C32—C31	114.56 (19)
C12—C13—H13B	108.9	C33—C32—H32A	108.6
H13A—C13—H13B	107.7	C31—C32—H32A	108.6
C13—C14—C15	114.0 (2)	C33—C32—H32B	108.6
C13—C14—H14A	108.8	C31—C32—H32B	108.6
C15—C14—H14A	108.8	H32A—C32—H32B	107.6
C13—C14—H14B	108.8	C32—C33—C34	113.5 (2)
C15—C14—H14B	108.8	C32—C33—H33A	108.9
H14A—C14—H14B	107.7	C34—C33—H33A	108.9
C16—C15—C14	115.3 (2)	C32—C33—H33B	108.9
C16—C15—H15A	108.5	C34—C33—H33B	108.9
C14—C15—H15A	108.5	H33A—C33—H33B	107.7
C16—C15—H15B	108.5	C33—C34—H34A	109.5
C14—C15—H15B	108.5	C33—C34—H34B	109.5
H15A—C15—H15B	107.5	H34A—C34—H34B	109.5
C15—C16—C17	113.7 (2)	C33—C34—H34C	109.5
C15—C16—H16A	108.8	H34A—C34—H34C	109.5
C17—C16—H16A	108.8	H34B—C34—H34C	109.5
			
C7—N1—N2—C9	161.19 (16)	C13—C14—C15—C16	−71.2 (3)
Ni1—N1—N2—C9	−21.57 (19)	C14—C15—C16—C17	−177.2 (2)
C24—N3—N4—C26	159.67 (17)	C23—C18—C19—C20	1.0 (3)
Ni1—N3—N4—C26	−22.02 (19)	C24—C18—C19—C20	178.05 (17)
C6—C1—C2—C3	1.3 (3)	C18—C19—C20—C21	−0.2 (3)
C7—C1—C2—C3	179.60 (18)	C19—C20—C21—C22	0.3 (3)
C1—C2—C3—C4	−0.6 (3)	C20—C21—C22—C23	−1.1 (3)
C2—C3—C4—C5	0.1 (3)	C21—C22—C23—C18	1.9 (3)
C3—C4—C5—C6	−0.4 (3)	C19—C18—C23—C22	−1.8 (3)
C4—C5—C6—C1	1.2 (3)	C24—C18—C23—C22	−178.93 (18)
C2—C1—C6—C5	−1.7 (3)	N4—N3—C24—C18	−169.77 (15)
C7—C1—C6—C5	−179.93 (18)	Ni1—N3—C24—C18	12.2 (3)
N2—N1—C7—C1	−173.58 (15)	N4—N3—C24—C25	7.7 (2)
Ni1—N1—C7—C1	9.7 (3)	Ni1—N3—C24—C25	−170.32 (13)
N2—N1—C7—C8	5.9 (2)	C19—C18—C24—N3	36.4 (3)
Ni1—N1—C7—C8	−170.90 (14)	C23—C18—C24—N3	−146.61 (18)
C2—C1—C7—N1	38.0 (3)	C19—C18—C24—C25	−141.20 (18)
C6—C1—C7—N1	−143.77 (19)	C23—C18—C24—C25	35.8 (3)
C2—C1—C7—C8	−141.45 (19)	N3—N4—C26—S3	2.3 (2)
C6—C1—C7—C8	36.8 (3)	N3—N4—C26—S4	−176.86 (13)
N1—N2—C9—S1	0.3 (2)	Ni1—S3—C26—N4	14.51 (18)
N1—N2—C9—S2	−179.64 (13)	Ni1—S3—C26—S4	−166.32 (10)
Ni1—S1—C9—N2	16.87 (17)	C27—S4—C26—N4	7.20 (19)
Ni1—S1—C9—S2	−163.16 (10)	C27—S4—C26—S3	−172.01 (11)
C10—S2—C9—N2	10.39 (19)	C26—S4—C27—C28	−78.59 (18)
C10—S2—C9—S1	−169.58 (11)	S4—C27—C28—C29	175.22 (15)
C9—S2—C10—C11	−78.20 (19)	C27—C28—C29—C30	178.15 (19)
S2—C10—C11—C12	−174.06 (18)	C28—C29—C30—C31	−174.7 (2)
C10—C11—C12—C13	−177.1 (2)	C29—C30—C31—C32	177.5 (2)
C11—C12—C13—C14	78.4 (3)	C30—C31—C32—C33	−174.7 (2)
C12—C13—C14—C15	−171.3 (2)	C31—C32—C33—C34	179.3 (2)

## References

[bb1] Begum, K., Begum, S., Sheikh, C., Miyatake, R. & Zangrando, E. (2020). *Acta Cryst.* E**76**, 692–696. 10.1107/S205698902000506X PMC7199265 32431934

[bb2] Begum, M. S., Das, D., Zangrando, E., Rahman, S., Alodhayb, A., Begum, M. K., Sheikh, C. M., Miyatake, R., Howlader, M. B. H., Karim, M. R. & Chowdhury, M. B. (2023). *J. Mol. Struct.* **1277**, 134808.

[bb3] Begum, M. S., Zangrando, E., Howlader, M. B. H., Sheikh, M. C., Miyatake, R., Hossain, M. M., Alam, M. M. & Hasnat, M. A. (2016). *Polyhedron*, **105**, 56–61.

[bb4] Begum, M. S., Zangrando, E., Sheikh, M. C., Miyatake, R. & Hossain, M. M. (2015). *Acta Cryst.* E**71**, o265–o266. 10.1107/S205698901500568X PMC4438792 26029448

[bb5] Begum, M. S., Zangrando, E., Sheikh, M. C., Miyatake, R., Howlader, M. B. H., Rahman, M. N. & Ghosh, A. (2017). *Transit. Met. Chem.* **42**, 553–563.

[bb6] Boshaala, A., Said, M. A., Assirey, E. A., Alborki, Z. S., AlObaid, A. A., Zarrouk, A. & Warad, I. (2021). *J. Mol. Struct.* **1238**, 130461.

[bb7] Boshaala, A., Yamin, B. M., Amer, Y. O. B., Ghaith, G. S. H., Almughery, A. A., Zarrouk, A. & Warad, I. (2021). *J. Mol. Struct.* **1224**, 129207.

[bb9] Bin Break, K. M., Tahir, M. I. M., Crouse, K. A. & Khoo, T.-J. (2013). *Bioinorg. Chem. Appl.* 362513. 10.1155/2013/362513 PMC3844202 24319401

[bb8] Brandenburg, K. (1999). *DIAMOND.* Crystal Impact GbR, Bonn, Germany.

[bb10] Cavalcante, C. de Q. O., Arcanjo, D. da S., da Silva, G. G., de Oliveira, D. M. & Gatto, C. C. (2019). *New J. Chem.* **43**, 11209–11221.

[bb11] Chan, M.-H. E., Crouse, K. A., Tahir, M. I. M., Rosli, R., Umar-Tsafe, N. & Cowley, A. R. (2008). *Polyhedron*, **27**, 1141–1149.

[bb12] Chew, K. B., Tarafder, M. T. H., Crouse, K. A., Ali, A. M., Yamin, B. M. & Fun, H. K. (2004). *Polyhedron*, **23**, 1385–1392.

[bb13] Crouse, K. A., Chew, K. B., Tarafder, M. T. H., Kasbollah, A., Ali, A. M., Yamin, B. M. & Fun, H. K. (2004). *Polyhedron*, **23**, 161–168.

[bb14] Farrugia, L. J. (2012). *J. Appl. Cryst.* **45**, 849–854.

[bb15] Flörke, U. & Boshaala, A. (2016). *CSD Communication* (refcode UWATOD). CCDC, Cambridge, England. https://doi.org/10.5517/ccdc.csd.cc1mgkpb

[bb16] Higashi, T. (1995). *ABSCOR*. Rigaku Corporation, Tokyo, Japan.

[bb17] How, F. N.-F., Crouse, K. A., Tahir, M. I. M., Tarafder, M. T. H. & Cowley, A. R. (2008). *Polyhedron*, **27**, 3325–3329.

[bb18] How, F. N.-F., Watkin, D. J., Crouse, K. A. & Tahir, M. I. M. (2007). *Acta Cryst.* E**63**, o2912.

[bb19] Howlader, M. B. H., Begum, M. S., Sheikh, M. C., Miyatake, R. & Zangrando, E. (2015). *Acta Cryst.* E**71**, m26–m27. 10.1107/S2056989015000328 PMC4384570 25878838

[bb20] Islam, M. A.-A. A. A., Sheikh, M. C., Alam, M. S., Zangrando, E., Alam, M. A., Tarafder, M. T. H. & Miyatake, R. (2014). *Transition Met. Chem.* **39**, 141–149.

[bb21] Khan, S. S., Howlader, M. B. H., Sheikh, M. C., Miyatake, R. & Zangrando, E. (2023). *Acta Cryst.* E**79**, 714–717. 10.1107/S2056989023005935 PMC10439407 37601399

[bb22] Nanjundan, N., Narayanasamy, R., Geib, S., Velmurugan, K., Nandhakumar, R., Balakumaran, M. D. & Kalaichelvan, P. T. (2016). *Polyhedron*, **110**, 203–220.

[bb23] Rigaku (2018). *RAPID-AUTO*. Rigaku Corporation, Tokyo, Japan.

[bb24] Shan, S., Wang, S.-H., Tian, Y.-L., Wang, W.-L. & Xu, Y.-L. (2008). *Acta Cryst.* E**64**, o1015. 10.1107/S160053680801283X PMC2961396 21202540

[bb25] Sheldrick, G. M. (2015*a*). *Acta Cryst.* A**71**, 3–8.

[bb26] Sheldrick, G. M. (2015*b*). *Acta Cryst.* C**71**, 3–8.

[bb27] Yang, P., Chen, H., Wang, Z.-Z., Zhang, L.-L., Zhang, D.-D., Shi, Q.-S. & Xie, X.-B. (2020). *J. Inorg. Biochem.* **213**, 111248. 10.1016/j.jinorgbio.2020.111248 33011623

[bb28] Zangrando, E., Islam, M. T., Islam, M. A.-A. A. A., Sheikh, M. C., Tarafder, M. T. H., Miyatake, R., Zahan, R. & Hossain, M. A. (2015). *Inorg. Chim. Acta*, **427**, 278–284.

